# Dapagliflozin use in tolvaptan-treated patients with ADPKD: exploring renal outcomes in a retrospective study

**DOI:** 10.1093/ckj/sfaf269

**Published:** 2025-09-03

**Authors:** Ryunosuke Nakajima, Shun Minatoguchi, Ryosuke Umeda, Shigehisa Koide, Midori Hasegawa, Hiroki Hayashi, Naotake Tsuboi

**Affiliations:** Department of Nephrology, Fujita Health University School of Medicine, Toyoake, Japan; Department of Nephrology, Fujita Health University School of Medicine, Toyoake, Japan; Department of Nephrology, Fujita Health University School of Medicine, Toyoake, Japan; Department of Nephrology, Fujita Health University School of Medicine, Toyoake, Japan; Department of Nephrology, Fujita Health University School of Medicine, Toyoake, Japan; Department of Nephrology, Fujita Health University School of Medicine, Toyoake, Japan; Department of Nephrology, Fujita Health University School of Medicine, Toyoake, Japan

**Keywords:** ADPKD, dapagliflozin, polycystic kidney disease, SGLT2 inhibitor, tolvaptan

## Abstract

**Background:**

Despite the widespread use of sodium-glucose cotransporter 2 inhibitors (SGLT2i) in the management of chronic kidney disease, their role in autosomal dominant polycystic kidney disease (ADPKD) remains unclear.

**Methods:**

This observational study evaluated the efficacy of the SGLT2i dapagliflozin in patients with ADPKD receiving tolvaptan. The primary outcome was the chronic estimated glomerular filtration rate (eGFR) slope, modeled using a multivariable linear mixed-effect model; a within-group analysis was also performed using an interrupted time series approach.

**Results:**

A total of 48 patients receiving tolvaptan were analyzed (24 patients in the control group vs 24 patients in the dapagliflozin group). The mean follow-up duration was 649 ± 363 days across all patients. The chronic eGFR slope was –2.30 [95% confidence interval (CI) –3.47, –1.13] in the control group and –1.72 (95% CI –3.48, –0.03) mL/min/1.73 m^2^ per year in the dapagliflozin group (*P* = .595). In within-group analysis using an interrupted time series approach, the chronic eGFR slope changed from –2.34 (95% CI –3.39, –1.30) to –1.14 (95% CI –2.68, 0.40) mL/min/1.73 m^2^ per year following dapagliflozin initiation (*P* = .191). No serious adverse events were observed during the follow-up period.

**Conclusions:**

Although no statistically significant differences were observed, both between- and within-group analyses showed a numerically slower decline in eGFR with dapagliflozin. Importantly, no evidence of harm was observed. These findings may contribute to ongoing discussions regarding the potential role of SGLT2i in ADPKD.

## GRAPHICAL ABSTRACT

**GRAPHICAL ABSTRACT:**
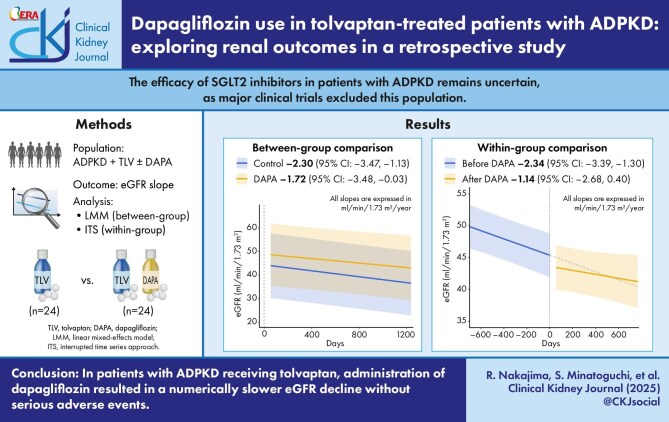

KEY LEARNING POINTS **What was known:**

- Despite the widespread use of tolvaptan, a key drug in the management of autosomal dominant polycystic kidney disease (ADPKD), the disease remains a major cause of end-stage kidney disease.
- Major clinical trials have shown that sodium-glucose cotransporter 2 inhibitors (SGLT2i) also suppress the progression of both diabetic and non-diabetic chronic kidney disease; however, these trials excluded patients with ADPKD, leaving the effects of SGLT2i in this population unclear.
- Some case studies have suggested that dapagliflozin may worsen the GFR slope or increase total kidney volume in patients with ADPKD.

**This study adds:**

- Among patients with ADPKD receiving tolvaptan, the addition of dapagliflozin resulted in a numerically slower eGFR decline compared with the control group.
- A within-group analysis suggested a less steep decline in eGFR following dapagliflozin initiation.
- Favorable changes in hemoglobin, hematocrit and uric acid levels were observed.

**Potential impact:**

- Dapagliflozin may offer potential renoprotective benefits in patients with ADPKD receiving tolvaptan.

## INTRODUCTION

Sodium-glucose cotransporter 2 inhibitors (SGLT2i) have been shown to exert renal and cardioprotective effects in diabetic kidney disease [1]. Major clinical trials, including the DAPA-CKD trial (Dapagliflozin and Prevention of Adverse Outcomes in Chronic Kidney Disease) and the EMPA-KIDNEY trial (Study of Heart and Kidney Protection with Empagliflozin), have clearly demonstrated that dapagliflozin and empagliflozin slow the progression of non-diabetic chronic kidney disease (CKD) [2, 3]. However, both trials excluded patients with autosomal dominant polycystic kidney disease (ADPKD), leaving the effects of SGLT2i in this population unclear [4, 5].

Although tolvaptan, a selective vasopressin V2 receptor antagonist, is widely used as a key therapy for ADPKD [6], the disease continues to be a major cause of kidney failure. Tolvaptan can slow the increase in total kidney volume (TKV) and the decline in kidney function, but its therapeutic effect remains modest in many cases. In this context, SGLT2i have attracted interest as a potential therapeutic option for patients with ADPKD.

However, recent case reports have raised concerns regarding the use of SGLT2i in patients with ADPKD. Morioka and Nakatani *et al.* reported that administration of dapagliflozin was associated with a decline in estimated glomerular filtration rate (eGFR) and an increase in TKV [7, 8]. In their retrospective single-arm case series, eGFR decreased from 47.9 to 40.8 mL/min/1.73 m^2^ and height-adjusted TKV increased from 599 to 617 mL/m in 20 patients with ADPKD treated with dapagliflozin 10 mg over an average follow-up of 102 ± 20 days.

In contrast, we previously reported a case of a patient with ADPKD receiving tolvaptan who was successfully treated with dapagliflozin for nearly 2 years without adverse outcomes [9]. Similarly, Yoshimoto *et al.* observed a less steep eGFR slope in seven patients with ADPKD treated with dapagliflozin in a retrospective study [10]. These previous case studies, however, lacked control groups, making it difficult to distinguish the effects of SGLT2i from the natural progression of the disease or other confounding factors.

Building on these findings, a more recent open-label, randomized, controlled crossover study by Uchiyama *et al.* provided preliminary evidence on the effects of dapagliflozin in patients with ADPKD. In this 6-month study involving 27 patients, dapagliflozin treatment attenuated the eGFR slope compared with the control period. While the findings are suggestive, the small sample size and short follow-up period limit the generalizability of the results [11]. Therefore, additional evidence from clinical studies is needed to complement these findings. This study aimed to evaluate the efficacy and safety of dapagliflozin in patients with ADPKD, providing further insight into its potential role in clinical practice.

## MATERIALS AND METHODS

### Study design and participants

A retrospective observational study was conducted to evaluate the effectiveness of dapagliflozin in patients with ADPKD who were receiving tolvaptan at Fujita Medical University Hospital and Tokoname Municipal Hospital, two tertiary referral hospitals in Japan. Patients were selected based on their treatment records between 1 August 2021 and 31 October 2024. They were classified into two groups: the dapagliflozin group, comprising patients who newly initiated dapagliflozin during this period, and the control group, consisting of patients who did not receive dapagliflozin. Dapagliflozin was initiated for the management of CKD in these patients, in accordance with its approved indication. This was not regarded as off-label use. Tolvaptan was prescribed based on the indication criteria under the public health insurance system in Japan, which require a TKV ≥750 mL and an annual increase in TKV ≥5%. However, the final decision to initiate treatment was at the discretion of the treating physician, considering the overall clinical context, including a family history of progressive disease. To ensure a more homogeneous study population, we excluded patients with CKD stage G5 at time zero. Time zero was defined separately for each group to align the starting point for follow-up. For the dapagliflozin group, it was the date of dapagliflozin initiation. For the control group, it was either 1 August 2021 (when dapagliflozin became commercially available) or the date of tolvaptan initiation, whichever occurred later. The follow-up period continued until 31 January 2025. If either dapagliflozin or tolvaptan was discontinued for any reason, subsequent data were excluded from the analysis. Additionally, patients with a follow-up period of <12 weeks were also excluded from the analysis.

### Outcomes

The primary outcome was the eGFR slope during the follow-up period, estimated as the chronic eGFR slope. To calculate the chronic eGFR slope, data from the first 2 months after time zero were excluded to minimize the influence of early treatment effects, including those potentially associated with initiation or dose titration of tolvaptan, as well as with dapagliflozin administration. Although no universally accepted cutoff exists, excluding the early phase of treatment is a common approach in observational studies evaluating eGFR slope. Based on clinical considerations, we selected a 2-month window to capture the more stable phase of renal function. To evaluate the short-term impact of dapagliflozin administration, the initial dip in eGFR was assessed, defined as the lowest value observed between 2 and 8 weeks after time zero. Additionally, a within-group analysis was performed to compare the eGFR slope before and after dapagliflozin initiation.

Secondary outcomes included changes in TKV and hemoglobin (Hb), hematocrit (Hct) and uric acid (UA) levels, which were compared between the two groups. TKV was assessed using abdominal computed tomography images obtained during routine clinical practice, and measured by trained radiologists at each institution using the planimetry methods [12]. Demographic, clinical and laboratory data collected during the follow-up period were extracted from patients’ medical records.

### Ethical considerations

The study was approved by the Ethics Committee of Fujita Medical University (ID: HM23-089) and conducted in accordance with the principles of the Declaration of Helsinki. An opt-out approach to informed consent was used based on the approval of the ethics board because of the retrospective nature of this study.

### Statistical analyses

Baseline characteristics were compared between groups using standardized mean differences (SMD), with an SMD <0.1 considered to indicate good balance. Continuous variables were reported as means and standard deviations, while categorical variables as numbers and percentages.

To estimate changes in eGFR over the follow-up period, we applied a multivariable linear mixed-effects model (LMM) with random intercepts and random slopes. Fixed effects included dapagliflozin prescription, day, interaction term between dapagliflozin and day, renin–angiotensin system inhibitor, body mass index (BMI), tolvaptan dose, tolvaptan duration prior to time zero, hypertension, Hb, albumin (Alb), UA and TKV. These covariates were selected based on prior clinical knowledge and their potential roles as confounders affecting both treatment assignment and kidney function decline in patients with ADPKD [13]. Each patient was modeled as a random effect to account for individual variability in baseline eGFR and its trajectory. To estimate the eGFR slope before and after dapagliflozin initiation, we performed an interrupted time series (ITS) analysis using a linear missed-effects model. This model included dapagliflozin prescription, day and the interaction term between them as fixed effects, with patients modeled as random effects. The ITS approach allowed us to evaluate changes in the trend of eGFR slope associated with the initiation of dapagliflozin.

Paired comparisons of relevant clinical parameters were conducted using the Wilcoxon rank-sum test as appropriate. A *P*-value of <.05 was considered statistically significant. All analyses were conducted using R software, version 4.4.0 (The Comprehensive R Archive Network: https://cran.r-project.org). The “nlme” and “lme4” packages were used for mixed-effects modeling [14, 15].

## RESULTS

**Figure 1:**
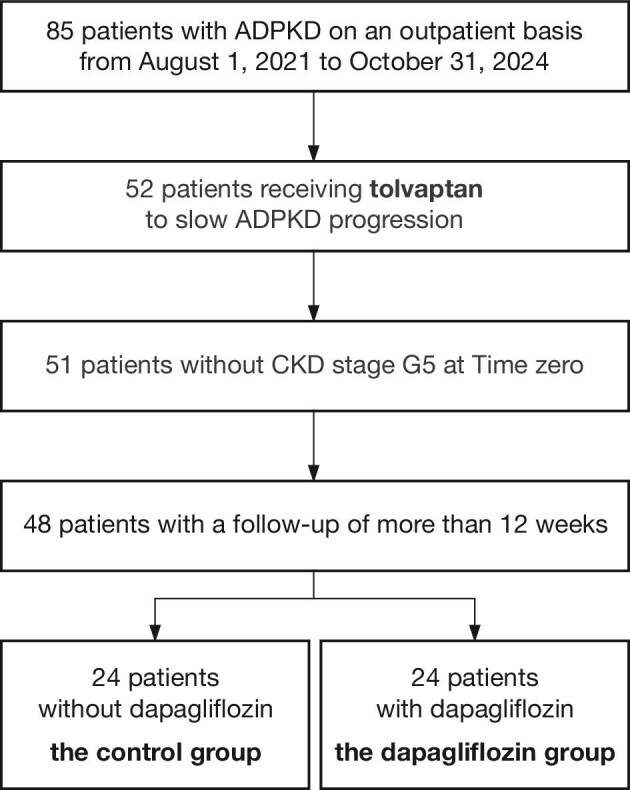
Study flow chart.

From 1 August 2021 to 31 December 2024, we identified 85 patients with ADPKD, including 52 patients receiving tolvaptan to slow disease progression. After excluding patients with CKD stage G5, and those with a short follow-up period, a total of 48 patients were included in the analysis. Of these, 24 patients were newly prescribed dapagliflozin, while the remaining 24 patients who continued to receive tolvaptan treatment without dapagliflozin (Fig. 1). Most cases were from Fujita Medical University Hospital, while an additional four patients from Tokoname Municipal Hospital, all of whom received dapagliflozin, were also included. The mean follow-up duration was 649 ± 363 days across all patients. A small number of patients discontinued dapagliflozin or tolvaptan during follow-up, and subsequent data were excluded from the eGFR slope analysis (Supplementary data, Table S1).

Table 1 summarizes the baseline characteristics of the study population. The mean age was 50 ± 12 years in the control group and 47 ± 8 years in the dapagliflozin group. The mean BMI was 22.3 ± 2.9 kg/m^2^ in the control group and 21.7 ± 2.5 kg/m^2^ in the dapagliflozin group. The mean TKV was 1491 ± 800 mL in the control group and 1575 ± 970 mL in the dapagliflozin group. Tolvaptan at the maximum approved dose of 120 mg/day was used in 50% of those in the control group and in 91.7% of patients in the dapagliflozin group. The mean duration of tolvaptan use prior to time zero was shorter in the control group (1.21 ± 2.01 years) than in the dapagliflozin group (4.42 ± 2.65 years). None of the groups included patients with diabetes or proteinuria, and all patients had preserved cardiac function with normal left ventricular ejection fraction. The eGFR was lower in the dapagliflozin group (52.4 ± 22.1 vs 44.8 ± 19.2 mL/min/1.73 m^2^).

**Table 1:** Baseline characteristics of study participants.

	Control	Dapagliflozin	SMD
*n*	24	24	
Male	11 (45.8)	11 (45.8)	<0.001
Age, years	50 ± 12	47 ± 8	0.288
BMI, kg/m^2^	22.3 ± 2.9	21.7 ± 2.5	0.209
Mayo classification			0.428
1B	7 (29.2)	6 (25.0)	
1C	12 (50.0)	9 (37.5)	
1D	3 (12.5)	7 (29.2)	
1E	2 (8.3)	2 (8.3)	
TKV, mL	1491 ± 800	1575 ± 970	0.095
Tolvaptan dose, %			1.072
60 mg/day	5 (20.8)	0 (0.0)	
90 mg/day	7 (29.2)	2 (8.3)	
120 mg/day	12 (50.0)	22 (91.7)	
Tolvaptan duration prior to time zero, years	1.21 ± 2.01	4.42 ± 2.65	1.364
Smoke	10 (41.7)	3 (13.6)	0.660
HTN	20 (83.3)	15 (62.5)	0.482
LVEF, %	60.58 ± 3.32	59.18 ± 3.92	0.386
DM	0 (0.0)	0 (0.0)	<0.001
RASi	16 (66.7)	15 (62.5)	0.087
Diuretics	1 (4.2)^a^	0 (0.0)	0.295
CCB	13 (54.2)	3 (12.5)	0.985
BUN, mg/dL	19.1 ± 6.5	20.5 ± 8.7	0.178
eGFR, mL/min/1.73 m^2^	52.4 ± 22.1	44.8 ± 19.2	0.367
CKD stage			0.481
G2	11 (45.8)	6 (25.0)	
G3a	4 (16.7)	7 (29.2)	
G3b	4 (16.7)	4 (16.7)	
G4	5 (20.8)	7 (29.2)	
UPCR, mg/gCr	128.9 ± 157.6	144.3 ± 182.8	0.090
Alb, g/dL	4.0 ± 0.3	4.2 ± 0.4	0.616
UA, mg/dL	5.4 ± 1.3	5.9 ± 1.0	0.458
Hb, g/dL	12.5 ± 1.0	13.1 ± 1.6	0.456
Hct, %	37.6 ± 3.0	40.1 ± 5.0	0.622
Glu, mg/dL	93 ± 15	93 ± 13	0.034

Data are presented as *n* (%) or mean ± standard deviation.

a One patient in the control group was receiving indapamide 1 mg/day as part of a long-standing antihypertensive regimen.

HTN, hypertension; LVEF, left ventricular ejection fraction; DM, diabetes mellitus; RASi, renin angiotensin system inhibitors; CCB, calcium channel blockers; BUN, blood urea nitrogen; UPCR, urine protein–creatinine ratio; Glu, glucose.

**Figure 2:**
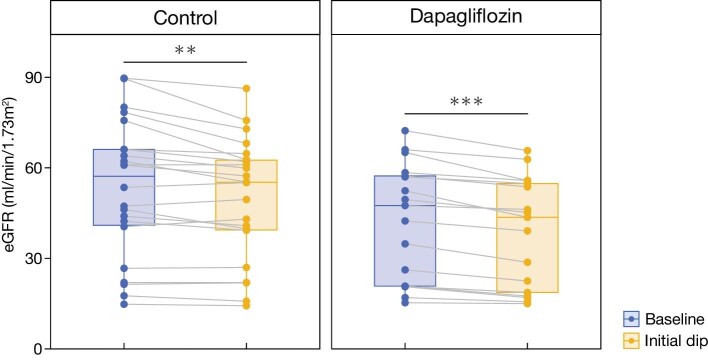
Initial eGFR dip during the first 2 months. Paired eGFR values at baseline and at the initial dip are shown. Due to missing data, 22 cases in the control group and 17 cases in the dapagliflozin group were included. The initial dip was defined as the lowest eGFR value recorded between 2 and 8 weeks after time zero. ^**^P < .01, ^***^P < .001 (Wilcoxon signed-rank test).

**Figure 3:**
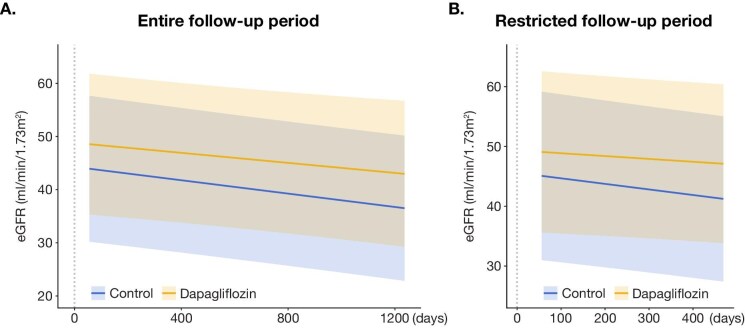
Between-group analysis of eGFR slope using LMM (control vs dapagliflozin). The chronic eGFR slope was estimated using the linear mixed-effects model with all available eGFR data throughout the follow-up period (**A**, entire follow-up period), as well as with data truncated to the mean follow-up period of the dapagliflozin group (**B**, restricted follow-up period).

In total, 764 eGFR measurements were obtained during the follow-up period (Supplementary data, Fig. S1). The mean follow-up duration for eGFR was 829 ± 406 days in the control group and 470 ± 192 days in the dapagliflozin group. During the follow-up period, no patient in either group died or initiated dialysis. Using the available data during the initial 2–8 weeks of treatment, including 22 patients in the control group and 17 in the dapagliflozin group, the initial eGFR dip was calculated (Fig. 2). In the dapagliflozin group, eGFR declined by –9.6 ± 5.4%, from 42.5 ± 19.3 to 38.7 ± 18.2 mL/min/1.73 m^2^ (*P* < .001). In the control group, the change was –5.2 ± 6.7%, from 53.2 ± 22.9 to 49.8 ± 20.2 mL/min/1.73 m^2^ (*P* = .0025). Although the initial eGFR dip was more substantial in the dapagliflozin group than in the control group (*P* = .04), the decline was not sustained. LMM-based estimation showed a numerically less steep eGFR slope in the dapagliflozin group, although the difference was not statistically significant. In the crude model, the interaction term between dapagliflozin and day was 0.00113 [95% confidence interval (CI) –0.00453, 0.0068; *P* = .694]. This corresponds to an estimated chronic eGFR slope over the entire follow-up period of –2.19 (95% CI –3.31, –1.06) in the control group and –1.77 (95% CI –3.50, –0.04) mL/min/1.73 m^2^ per year in the dapagliflozin group (Supplementary data, Table S2). In the adjusted model, the interaction term was 0.00157 (95% CI –0.00422, 0.00735; *P* = .595), corresponding to a chronic eGFR slope of –2.30 (95% CI –3.47, –1.13) in the control group and –1.72 (95% CI –3.48, –0.03) mL/min/1.73 m^2^ per year in the dapagliflozin group (Fig. 3, Table 2).

**Table 2:** Adjusted between-group analysis of eGFR slope using LMM (control vs dapagliflozin).

	Entire follow-up period		Restricted follow-up period	
Variable	Estimate (95% CI)	*P*-value	Estimate (95% CI)	*P*-value
(Intercept)	57.8 (–49.1, 165)	.288	58.6 (–47.6, 165)	.279
Day × Dapagliflozin	0.00157 (–0.00422, 0.00735)	.595	0.00455 (–0.00426, 0.0134)	.31
Day	–0.00629 (–0.0095, –0.00308)	.00013	–0.00931 (–0.0152, –0.00347)	.00187
Dapagliflozin	4.53 (–10.8, 19.9)	.554	3.74 (–11.8, 19.2)	.628
RASi	–2.39 (–18.2, 13.4)	.761	–2.81 (–18.4, 12.8)	.718
BMI	0.914 (–1.43, 3.26)	.435	0.792 (–1.54, 3.12)	.496
Tolvaptan dose	–0.00988 (–0.357, 0.337)	.954	0.0069 (–0.337, 0.351)	.968
Tolvaptan duration prior to time zero	–3.25 (–5.84, –0.659)	.0154	–3.34 (–5.91, –0.77)	.0123
HTN	–4.83 (–23.6, 14)	.606	–4.24 (–22.8, 14.3)	.646
Hb	0.802 (–4.09, 5.69)	.742	0.729 (–4.13, 5.58)	.763
Alb	2.7 (–16.3, 21.7)	.775	2.03 (–16.8, 20.9)	.828
UA	–4.95 (–10.5, 0.553)	.0765	–4.02 (–9.49, 1.44)	.144
TKV	–0.00688 (–0.0144, 0.00063)	.0716	–0.00728 (–0.0147, 0.00018)	.0555

RASi, renin angiotensin system inhibitors; HTN, hypertension.

As the follow-up period differed between the groups (829 ± 406 days in the control group and 470 ± 192 days in the dapagliflozin group), the estimation may have been biased. Therefore, to reduce potential bias, we also conducted a supportive analysis by truncating the follow-up period to match the mean duration of the dapagliflozin group (470 days). Even after truncation, a similar trend toward a more gradual eGFR slope was observed. In the crude model using the restricted follow-up dataset, the interaction term between dapagliflozin and day was 0.00433 (95% CI –0.00446, 0.0131; *P* = .333). This corresponds to a chronic eGFR slope of –3.37 (95% CI –5.49, –1.25) in the control group and –1.79 (95% CI –4.19, 0.60) mL/min/1.73 m^2^ per year in the dapagliflozin group (Supplementary data, Table S2). In the adjusted model, the interaction term was 0.00455 (95% CI –0.00426, 0.0134; *P* = .31), corresponding to a chronic eGFR slope of –3.40 (95% CI –5.52, –1.27) in the control group and –1.74 (95% CI –4.14, 0.66) mL/min/1.73 m^2^ per year in the dapagliflozin group (Fig. 3, Table 2).

**Figure 4:**
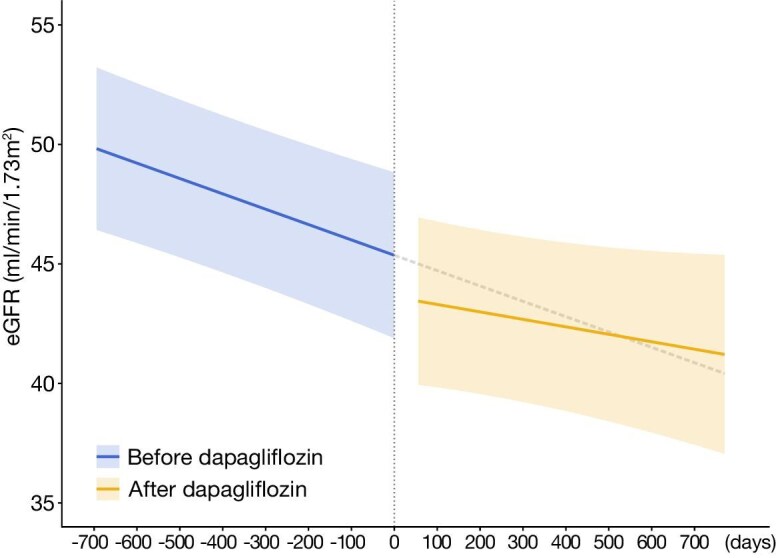
Within-group analysis of eGFR slope using ITS with LMM (pre-post dapagliflozin). In the dapagliflozin group, the eGFR slope was estimated using an interrupted time series approach with a linear mixed-effects model. The model incorporated data from 2 years before time zero (before dapagliflozin) and from 2 months after time zero to the end of follow-up (after dapagliflozin). The dotted line indicates the counterfactual eGFR slope.

To further investigate the effects of dapagliflozin on eGFR slope, we conducted a within-group analysis using an ITS design, comparing the eGFR slope before and after dapagliflozin initiation. eGFR data from the 2 years prior to dapagliflozin initiation were used. An LMM was applied to estimate the eGFR slope, showing a slight attenuation in the eGFR slope following dapagliflozin administration, although the change was not statistically significant (Fig. 4, Table 3). The interaction term between dapagliflozin and day was 0.0033 (95% CI –0.00163, 0.00823; *P* = .191), indicating that the estimated chronic eGFR slope was –2.34 (95% CI –3.39, –1.30) before dapagliflozin administration and –1.14 (95% CI –2.68, 0.40) mL/min/1.73 m^2^ per year after initiation.

**Table 3:** Within-group analysis of eGFR slope using ITS with LMM (pre-post dapagliflozin).

Variable	Estimate (95% CI)	*P*-value
(Intercept)	45.4 (41.9, 48.8)	<.001
Day × Dapagliflozin	0.0033 (–0.00163, 0.00823)	.191
Day	–0.00642 (–0.00929, –0.00355)	<.001
Dapagliflozin	–1.75 (–3.45, –0.0429)	.0452

Next, we evaluated changes in TKV. Due to missing data, paired comparisons were possible for 27 cases (16 in the control group and 11 in the dapagliflozin group). The mean TKV before time zero was 1599 ± 890 mL and 1366 ± 984 mL in the control and the dapagliflozin groups, respectively, with measurements performed –304 ± 229 days and –231 ± 173 days relative to time zero. One year after time zero, the mean TKV increased to 1837 ± 1237 mL and 1497 ± 1097 mL in the control and the dapagliflozin groups, respectively. In both groups, TKV increased after time zero, and the increase appeared greater in the control group than in the dapagliflozin group. However, because the timing of TKV measurement varied widely, with baseline TKV scans performed before and after tolvaptan initiation depending on the case [9 of 24 patients (38%) in the control group and 18 of 24 patients (75%) in the dapagliflozin group had baseline TKV scans during tolvaptan use], a direct comparison of TKV change between groups may not be appropriate. The mean measurement dates after time zero were 519 ± 224 days in the control group and 326 ± 114 days in the dapagliflozin group. Furthermore, although baseline TKV data were available, values immediately preceding dapagliflozin initiation were not consistently collected, making it difficult to accurately evaluate the rate of TKV increase following treatment.

**Figure 5:**
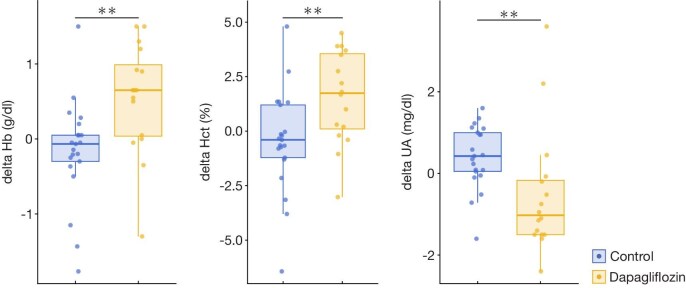
One-year changes in Hb, Hct and UA. One-year changes in Hb, Hct and UA in each group are shown for each group. ^**^P < .01 (Wilcoxon rank sum test).

We also assessed the changes in Hb, Hct and UA levels (Fig. 5). One year after time zero, significant increase in Hb and Hct levels were observed in the dapagliflozin group compared with the control group. Hb increased by 0.54 ± 0.74 g/dL in the dapagliflozin group versus an –0.16 ± 0.68 g/dL in the control group (*P* = .003). Similarly, Hct increased by 1.55 ± 2.13% in the dapagliflozin group versus –0.44 ± 2.34% in the control group (*P* = .009). Changes in UA levels were –0.52 ± 1.53 in the dapagliflozin group versus 0.41 ± 0.77 in the control group (*P* = .003).

Finally, we evaluated the safety and tolerability of adding dapagliflozin to standard treatment with tolvaptan (Supplementary data, Table S1). Among 27 patients who were newly prescribed dapagliflozin before excluding those with short follow-up, five patients (19%) discontinued the drug. Reasons for discontinuation included frequent urination (*n* = 2), nausea (*n* = 1), decreased blood pressure (*n* = 1) and liver function abnormalities (*n* = 1). No serious adverse events including cyst infection or urinary tract infection, were observed during the follow-up period.

## DISCUSSION

In the present observational study, we compared patients with ADPKD who received only tolvaptan and those who received both dapagliflozin and tolvaptan. Although no statistically significant improvement in the eGFR slope was observed, both the between-group and within-group analyses showed a numerically slower decline in eGFR with dapagliflozin. Importantly, no evidence of accelerated eGFR decline or serious adverse events was observed with dapagliflozin, suggesting that it did not result in apparent harm in this patient population. This finding is noteworthy given the current guideline [13], which is mainly based on animal studies and the absence of robust randomized controlled trials (RCTs), recommends against the use of SGLT2i in patients with ADPKD. In addition, the dapagliflozin group included slightly more patients with CKD stage G4, who are generally expected to have faster disease progression. Despite this, the eGFR slope in the dapagliflozin group was not worse than that in the control group, suggesting both tolerability and a possible renoprotective effect even in patients with more advanced CKD. Still, this should be interpreted with caution given the observational nature of the study.

Despite the widespread use of SGLT2i in treating patients with CKD over the past few years, their application in ADPKD remains controversial, with no established consensus. The uncertainty is compounded by conflicting results from preclinical studies in rodent models. For example, Wang *et al.* reported that phlorizin reduced the progression of cystic disease in a rat model of polycystic kidney disease, while Kapoor *et al.* observed that dapagliflozin induced osmotic diuresis, hyperfiltration and albuminuria, along with an increase in cyst volume [16, 17]. These divergent findings highlight the unclear efficacy and safety profile of SGLT2i in ADPKD. Moreover, clinical evidence for effectiveness of SGLT2i in patients with ADPKD remains limited to a few case reports and small-scale studies, which primarily rely on pre- and post-treatment comparisons without robust controls. A recent pilot RCT by Uchiyama *et al.* suggested that dapagliflozin may slow the decline in eGFR in patients with ADPKD. In their study of 27 patients over 6 months, the chronic eGFR slope calculated using both creatinine and cystatin C levels was 2.57 ± 7.88 mL/min/1.73 m^2^ per year with dapagliflozin compared with –5.65 ± 9.57 mL/min/1.73 m^2^ per year without dapagliflozin [11]. While these findings are encouraging, the small sample size and short follow-up limit their generalizability. Another recent observational study by Eswarappa *et al.* examined the effects of SGLT2i in a real-world cohort of US veterans with mild ADPKD [18]. Although the findings suggested potential renoprotective benefits, most patients were older and had comorbidities such as diabetes and cardiovascular disease. Moreover, tolvaptan was used in only one patient in their cohort. These characteristics differ from those of patients for whom tolvaptan is considered standard care, and thus the findings may offer limited insight into such ADPKD populations.

Currently, RCTs with limited sample sizes (*n* = 40–50) assessing the efficacy and safety of SGLT2i in patients with ADPKD over a 12-month period are underway (ClinicalTrials.gov identifiers: NCT06391450 and NCT05510115) [19], though results are not expected to be available in the near future. In the meantime, observational studies such as ours offer valuable real-world data to help bridge the evidence gap, as recently highlighted by Müller *et al.* in a review on SGLT2i in patients with ADPKD [20]. Although observational designs have inherent limitations, their findings can support clinical decision-making until more definitive RCT data become available. While our study did not demonstrate statistically significant changes, it still provides meaningful insights in a context where the use of SGLT2i in patients with ADPKD is under debate. Although the lack of statistical significance limits definitive conclusions, a numerically slower decline in eGFR was observed with dapagliflozin treatment across multiple analyses. Additionally, favorable changes in anemia and UA levels were observed, which are consistent with the known effects of SGLT2i, particularly given that anemia improvement is recognized as a key renoprotective mechanisms of SGLT2i [21, 22].

Moreover, as part of an additional analysis, we applied an interrupted time series approach within the dapagliflozin group and observed a numerically less steep eGFR slope after dapagliflozin initiation. Notably, GFR trajectories in patients with ADPKD are often nonlinear, with a period of relative stability followed by accelerating decline [23]. Thus, even modest changes in slope may be clinically relevant and deserve attention. Given the phenotypic heterogeneity in patients with ADPKD, largely due to variable expression of the *PKD1* or *PKD2* genes, findings from the within-group comparisons may provide valuable complementary insights to those obtained from between-group analyses.

This study has several limitations. First, the follow-up duration was relatively short. Both the DAPA-CKD and EMPA-KIDNEY trial included 36 months of follow-up [2, 3], and it is generally recommended that eGFR slope be assessed over a period of at least 2–3 years [24]. Nonetheless, the duration in our study was sufficient to confirm that the initial reduction in eGFR following dapagliflozin initiation was not sustained. Second, the sample size was small, which limits statistical power and may result in underestimation of treatment effects. Third, due to the observational nature of this study and the use of routine clinical data, several important variables were unavailable or inconsistently collected, including scheduled TKV measurements, daily diuresis and urinary osmolarity, and genetic testing results. In addition, the duration of prior tolvaptan use differed between groups, which may have influenced eGFR slope comparisons despite adjustment. The distribution of tolvaptan dose also differed, with more patients receiving 120 mg/day in the dapagliflozin group, potentially contributing to residual confounding. Future studies, including RCTs, should aim to provide a more comprehensive assessment of the efficacy and safety for both tolvaptan and dapagliflozin in patients with ADPKD.

In conclusion, our observational study suggested that the addition of dapagliflozin to standard treatment with tolvaptan may be safe and could be associated with slower kidney function decline. Improvements in anemia were also observed, and within-group comparison indicated a possible attenuation in the eGFR slope. These findings may contribute to ongoing discussions regarding the potential role of SGLT2i in the management of ADPKD.

## Supplementary Material

sfaf269_Supplemental_File

## Data Availability

The data underlying this article will be shared on reasonable request to the corresponding author.
