# Supplementary material for: Dapagliflozin use in tolvaptan-treated patients with ADPKD: exploring renal outcomes in a retrospective study
Source: Clin Kidney J. 2025 Sep 3;18(9):sfaf269. doi: 10.1093/ckj/sfaf269 (PMC12461144; doi:10.1093/ckj/sfaf269)
Supplement: sfaf269_Supplemental_File [file sfaf269_supplemental_file.pdf]

Supplementary Material

Dapagliflozin Use in Tolvaptan-Treated Patients with ADPKD: Exploring Renal Outcomes in a Retrospective Study

Ryunosuke Nakajima†, Shun Minatoguchi†, Ryosuke Umeda, Shigehisa Koide, Midori Hasegawa, Hiroki Hayashi*, Naotake Tsuboi

†These authors contributed equally.
*Corresponding author

Supplementary Table S1. Summary of Cases who Discontinued Dapagliflozin or Tolvaptan and Corresponding Inclusion in eGFR Slope Analysis

	**Age** (yrs)	**Sex**	**Group**	**Discontinuation** (Drug / Reason)	**Date** (days)	**Included in eGFR slope analysis**
**Case 1**	44	Female	Dapagliflozin	Dapagliflozin / nausea	294	Included (until discontinuation)
**Case 2**	74	Male	Dapagliflozin	Dapagliflozin / Urinary frequency	28	Not included (follow-up < 12w)
**Case 3**	56	Male	Dapagliflozin	Dapagliflozin / Urinary frequency	28	Not included (follow-up < 12w)
**Case 4**	38	Male	Dapagliflozin	Dapagliflozin / Decrease in blood pressure	174	Included (until discontinuation)
**Case 5**	56	Male	Dapagliflozin	Dapagliflozin / Liver function abnormalities	63	Not included (follow-up < 12w)
**Case 6**	36	Female	Control	Tolvaptan / Liver function abnormalities	107	Included (until discontinuation)
**Case 7**	32	Male	Control	Tolvaptan / Liver function abnormalities	63	Included (until discontinuation)

Supplementary Table S2. Unadjusted Between-Group Analysis of eGFR Slope Using LMM (Control vs. Dapagliflozin)

	Entire follow-up period		Restricted follow-up period	
Variable	Estimate (95%CI)	p-value	Estimate (95%CI)	p-value
**(Intercept)**	51.4 (42.7, 60.2)	<0.00[illegible]	52.1 (43.1, 61.1)	<0.0001
**day×Dapagliflozin**	0.00113 (-0.00453, 0.0068)	0.694	0.00433 (-0.00446, 0.0131)	0.333
**Day**	-0.00599 (-0.00909, -0.00289)	0.000[illegible]	-0.00924 (-0.0151, -0.00341)	0.00197
**Dapagliflozin**	-7.56 (-20.2, 5.11)	0.236	-8.12 (-21.1, 4.87)	0.215

Abbreviation: eGFR, estimated glomerular filtration rate.

Supplementary Figure S1. All Measured eGFR Value in Each Group.

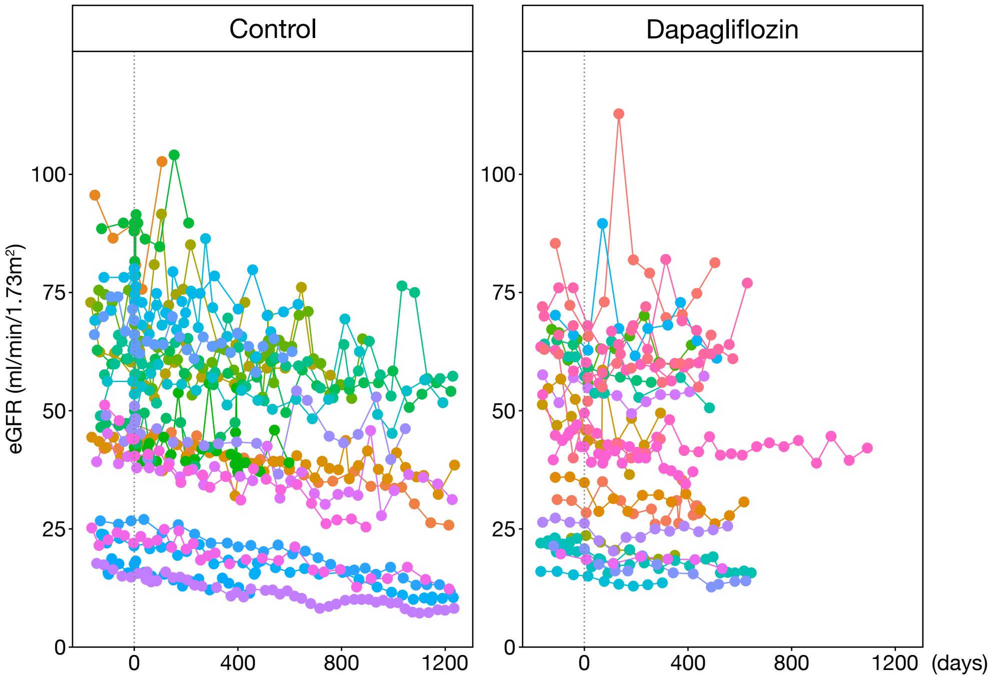

All Measured eGFR values for each group are shown. Each color represents an individual case (n = 48).

Abbreviation: eGFR, estimated glomerular filtration rate.
